# Time characteristics of the effect of alcohol cessation on the risk of stomach cancer – a meta-analysis

**DOI:** 10.1186/1471-2458-13-600

**Published:** 2013-06-20

**Authors:** Johan Jarl, Gawain Heckley, Julie Brummer, Ulf-G Gerdtham

**Affiliations:** 1 Health Economics & Management, Institute of Economic Research, Lund University, Box 117, Lund 22100, Sweden; 2 Center for Primary Health Care Research, Skåne University Hospital, Malmö, Lund University/Region Skåne, SE-20502, Malmö, Sweden; 3 Economics Department, Lund University, Box 117, Lund, 22100, Sweden; 4 Division of Health Economics, Department of Clinical Science, Lund University, Malmö University Hospital, SE-205 02, Malmö, Sweden

**Keywords:** Alcohol cessation, Stomach cancer, Meta-analysis

## Abstract

**Background:**

In the Bagnardi et al. (2001) meta-analysis, it was found that alcohol consumption increases the risk of stomach cancer (OR = 1.32 for heavy drinkers). However, it is unknown if drinking cessation reverses this alcohol-elevated risk.

**Methods:**

A systematic literature review was performed to provide the information for a meta-analysis where the dose-risk trend was estimated for years since drinking cessation and the risk of stomach cancer. A random effect generalised least squares model for trend estimation was used, employing study characteristics to control for heterogeneity.

**Results:**

Nineteen observational studies were identified in the literature review, of which five studies quantified duration of cessation and risk of stomach cancer, giving a total of 1947 cancer cases. No significant effect of drinking cessation on the risk of stomach cancer could be found (OR = 0.99 CI: 0.97-1.02).

**Conclusions:**

This result should be interpreted with caution due to the limited number of studies in this area. Recent findings suggest a link between heavy drinking and stomach cancer, especially gastric noncardia, but not for moderate drinking. Since all but one of the included studies in this meta-analysis failed to control for consumption level, the current study could not test if the risk decline following drinking cessation differs between moderate and high consumers.

## Background

Lifestyle and dietary habits have been extensively investigated for their relationship with gastric cancer, with a number of studies focusing on the role of alcohol intake [1-3]. Findings from studies examining the relationship between alcohol consumption and the risk of stomach cancer have been inconsistent. Many studies individually have failed to show a statistically significant increased risk of stomach cancer among drinkers [1,2,4-6]. However, some studies have demonstrated a positive association [3,7,8]. A meta-analysis of alcohol drinking and risk of stomach cancer by Bagnardi *et al*. [9] found a positive relationship with statistically significant increased relative risks of 1.07 (1.04, 1.10) for 25 g of pure alcohol per day, 1.15 (1.09, 1.22) for 50 g of pure alcohol per day and 1.32 (1.18, 1.49) for 100 g of pure alcohol per day. It should be noted that there are potentially different etiologies for distal and cardia gastric cancer which might lead to a different effect of alcohol consumption [3,10-12]. Based on the Bagnardi *et al*. study [9], stomach cancer was included in the Swedish cost-of-alcohol study [13]. More recently, however, Liu and Russell [14] stated that studies published after the Bagnardi meta-analysis [9] individually provide little support for a significant positive relationship. The latest word on the subject was a meta-analysis published in 2011, where a positive association was found for heavy drinking but not for moderate drinking [12]. It does appear that whether alcohol consumption raises the risk of stomach cancer will continue to be discussed over the coming years.

However, if stomach cancer, in any way, is caused by alcohol consumption, it is important for alcohol policy to have information on how fast, if at all, the increased risk from drinking falls after drinking cessation. This has implications for harm-reducing interventions as well as cost-effectiveness calculations [15]. Besides, stomach cancer has a high mortality rate [16], and focusing on prevention and intervention to reduce incidence rates is essential for fighting the disease in the short run. Information on the actual beneficial effect from quitting drinking alcohol could be helpful in motivational treatments for alcohol misuse.

The aim of this study was therefore to establish current knowledge regarding the effect of drinking cessation on the risk of stomach cancer. To this end, a meta-analysis was conducted based on relevant studies identified through a systematic literature review. This paper is structured as follows: First, the methods and materials are discussed, including the process by which studies were selected for the review and the data extraction process for the meta-analysis. This is then followed by the results of the meta-analysis, while the detailed results of the literature review are available as an Additional file 1. The final section discusses the results and conclusions.

## Methods

All studies were first identified through a systematic literature review. The purpose of the literature review was to both supply a complete collection of the available evidence for the meta-analysis of the trend in the relationship between time since drinking cessation and risk of gastric cancer without restrictions for study type and to give an indication of the quality and level of the current research. The process followed the PRISMA 2009 checklist (http://www.prisma-statement.org).

### Selection of studies

**Figure 1:**
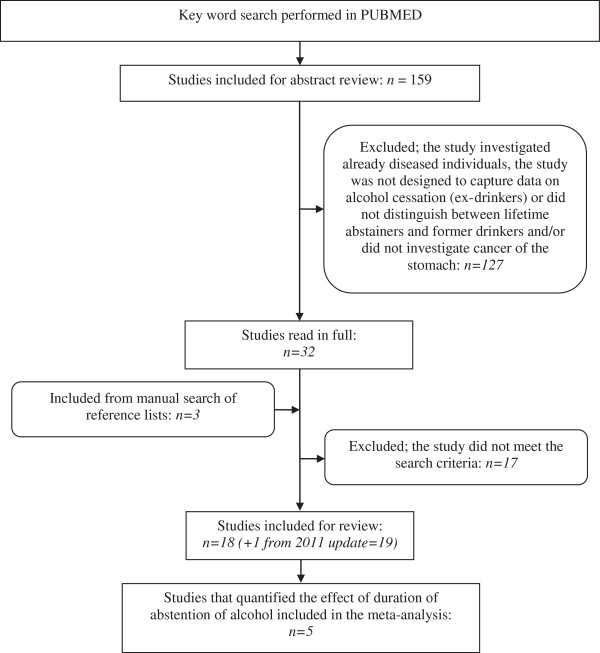
Flowchart of the systematic review process.

A literature search was conducted during June and July 2010 and updated in June 2011. Using the key words (“alcohol” OR “drinking” OR “lifestyle” OR “habit”) AND (“stomach cancer” OR “gastric cancer” OR “gastric adenocarcinomas”) AND (“cessation” OR “quit drinking” OR “ex drinker” OR “former drinker” OR “stop drinking” OR “stopping drinking” OR “abstainer” OR “abstinence” OR “consumption” OR “consumption level” OR “lifestyle” OR “habit” OR “protective”), searches were performed using the PUBMED database. These searches were supplemented by a review of papers cited in relevant articles. Studies were excluded for the following reasons: (1) the study was not published in English; (2) the study did not examine the risk of stomach cancer; or (3) the study did not investigate the risk of stomach cancer among former drinkers. Studies that investigated the relationship between alcohol consumption and the prognosis of already diseased individuals were not included. The results of the selection process are described by way of a flowchart (see Figure 1).

The requirements for inclusion in the meta-analysis were higher than for the systematic literature review. First, studies that did not present sufficient data on risks, confidence intervals, sample size, and/or the used reference category (current drinkers), or did not provide sufficient information to allow this to be calculated, were excluded. Second, if a data set was used in more than one study, only one of the studies was included.

### Data extraction

A large amount of data was extracted from the identified studies, including but not limited to study type, sample size, gender, age distribution, risk estimates with confidence intervals, and adjustment variables (see Additional file 1). Missing confidence intervals were calculated by the authors where necessary and possible. When several risk estimates were presented, the estimate with the largest sample size and/or most adjustment variables was used. When the estimated risk presented in a study was in relation to any other consumption group than current drinkers (most likely never-drinkers/abstainers), these were rescaled in relation to current drinkers by the authors. Using the approach set out in Hamling *et al*. [17], the standard errors and confidence intervals were re-calculated consistently for each rescaled risk estimate based on the adjusted results presented in the papers. In one case [18] were the Hamling method used to estimate expected number of cases and controls for each cessation group in order to achieve a common comparison group. Drinking cessation was commonly reported in categories, and the midpoints of the categories were used in the quantification. When a category was open-ended, the same interval width was applied as was for the previous category.

It is possible that recent quitters might be a group that stops drinking due to health-related problems (or even suspicion of stomach cancer) [19]. We therefore extracted information on the definition of a former drinker (length required since cessation). Information on risk decline for different consumption levels was also extracted when available, as stomach cancer is considered to be related to level of consumption [9,12]. Finally, whether smoking was controlled for in the identified studies was also collected, as smoking traditionally has been considered a risk factor [20].

The effect measure of interest was the odds ratio (OR), although some studies reported the relative risk (RR). When the prevalence of a disease is small, the OR coincides with the RR [21].

### Statistical analyses

In order to find whether a common trend exists amongst the current literature for the effect of years since drinking cessation on risk of disease, meta-analysis in the form of a Generalised Least Squares model (GLS) was utilised [22]. The approach estimates a linear trend using GLS after a covariance matrix for the log risks has been constructed that accounts for the covariances that exist between the relative risks calculated using a common reference group. Standard errors were used as weights as a proxy for study quality as is standard practice in the meta-analysis literature. The meta-regression took the form:

(1) $$y_{\mathrm{it}}=\beta_{1}t_{i}+\beta_{2}z_{i}+e_{\mathrm{it}}$$

where *y*_*it*_ is the log of risk of stomach cancer for study *i* with cessation duration *t*. *β*_*1*_*t*_*i*_ is the trend to be estimated of the change in cancer risk by alcohol cessation duration. *z* is an effect modifier, i.e. study characteristics that explain between-study heterogeneity (assuming no interaction between the trend and the effect modifiers). The effect modifiers can also influence the trend through interaction effects, such as *β*_*3*_*t*_*i*_*z*_*i*_. Effect modifiers therefore estimate a different intercept (dummy) for those studies with a particular characteristic and/or a different slope (interaction term). The model goes through the origin by definition (reference category OR = 1, log(OR) = 0), so no intercept is estimated. In order to allow for a better fit by increasing the available information in the analysis, the data were first pooled [22]. Variables were extracted from the included studies that could help explain potential variance between studies, i.e. controls for heterogeneity (*z*). Fixed effects models assume that each included study estimated the same underlying effect [22]. The Q-test for heterogeneity was applied to the meta-estimations as proposed by Greenland & Longnecker [23], with the null hypothesis that the model is fitted correctly. If heterogeneity remains, random effects models will be estimated by including an additive random effect in the estimation equation [22]. This assumes there is additional statistical uncertainty that needs to be accounted for between the studies (within-group random variation).

The trend estimation was followed by a meta-analysis comparing the highest cessation category (dose) with current drinkers, a type of analysis sometimes known as “highest vs. lowest”. This analysis was presented in a forest plot with weights based on the standard errors. Heterogeneity was assessed by the I^2^-statistics, which is based on the Q-statistics above, adjusting for degrees of freedom [24]. All statistical analyses were conducted in Stata version 12, using the “glst” and “metan” commands.

## Results

Nineteen observational studies that estimated the reduction in risk of stomach cancer following drinking cessation were identified. The detailed results of the literature review are available as an Additional file 1, while this section focuses on the meta-analysis of identified studies. Five of the 19 identified studies quantified the risk impact of drinking cessation by duration (Table 1), three of which split their results by gender. One study showed a significantly lower risk after drinking cessation for men. Two of the five studies showed former drinkers having a significantly higher risk of stomach cancer at some time point following drinking cessation compared to current drinkers, while the other two studies showed an insignificantly higher risk at some point after drinking cessation.

**Table 1:** **Summarised data for meta-analysis: odds ratios of former drinkers by duration compared to current drinkers (with adjusted confidence intervals**)

**Study**	**Study type**	**Gender**	**Cases / Controls**	**Years since drinking cessation**	**Odds ratio**	**95% Confidence interval**		**Rescaled**^‡^
						**Lower bound**	**Upper bound**	
Osaza et al. 2007 [25]	Prospective cohort	Male	519/420639*	0	1.0	.	.	Yes
			15/7436*	2.5	1.3	0.8	2.1	
			11/7669*	10	0.7	0.4	1.4	
			8/5416*	20	0.7	0.3	1.4	
		Female	69/183648*	0	1.0	.	.	
			3/2676*	10	2.5	0.5	12.2	
			2/1679*	20	2.1	0.3	12.7	
Chow et al. 1999 [26]	Case–control	Male	161/212	0	1.0	.	.	Yes
			31/11	3	4.4	1.6	12.2	
			12/18	6.5	0.8	0.3	2.4	
			16/28	12	0.8	0.3	2.0	
		Female	35/36	0	1.0	.	.	
			12/2	3	7.3	0.8	69.7	
			1/7	6.5	0.2	0.0	9.4	
			3/11	12	0.3	0.0	2.0	
Unakami et al. 1989 [27]	Case–control	Male	691/105	0	1.0	.	.	No
			11/20	7.5	0.1	0.0	0.2	
		Female	105/30	0	1.0	.	.	
			2/2	7.5	0.3	0.0	4.1	
Munoz et al. 2001 [18]	Case–control	Male	76/68	0	1.0	.	.	Yes
			17/13	7.5	1.2	0.3	4.9	
			9/8	12.5	1.0	0.3	3.6	
			9/5	17.5	1.6	0.4	6.6	
			7/4	22.5	1.3	0.3	5.3	
De Stefani et al. 1990 [28]	Case–control	Male	65/169	0	1.0	0.0	0.0	No
			18/33	2	1.0	0.5	2.1	
			9/37	6.5	0.5	0.2	1.2	
			30/100	11.5	0.9	0.5	1.6	

* Person years. ‡ Yes if the comparison group in a specific study was changed by the authors in order to achieve a common reference group between studies.

Inoue *et al*. [29] did quantify the risk impact of drinking cessation by duration but did not present the number of cases and controls for the individual OR-calculations, and the results from this study could therefore not be included in the meta-analysis. Five studies were thus analysed in the meta-analysis [18,25-28], giving a total of 1947 cancer cases in the final dataset. It should also be noted that Ozasa [25] investigated alcohol influence on stomach cancer mortality, while the other included studies investigated the effect on morbidity.

A random effect linear model of the dose-risk relationship was estimated, see Models 1–4, Table 2. Model 4 is the preferred model as it explains relatively more of the between-study heterogeneity, and it is jointly significant, resulting in an OR of 0.994 (CI:0.966-1.023). Studies that failed to control for smoking had a significantly lower risk at time of cessation, and studies that failed to define former drinkers as having abstained for at least a year (to account for the sick quitter effect) had a significantly higher risk at the time of cessation, but controlling for these study characteristics in the meta-regression did not significantly improve the explanation of between-study heterogeneity (Model 4). A sensitivity analysis was conducted where one study was excluded at a time, without changes in significance level or any larger movements in the point estimate.

**Table 2:** **Meta**-**analysis trend estimates of risk of stomach cancer after alcohol cessation**

	**Random effects meta analysis**			
**Explanatory variables:**	**Model 1**^**a**^	**Model 2**	**Model 3**	**Model 4**
Trend in OR	0.982*	0.988	0.990	0.994
Proportion in study male		0.915		
Study did not control for smoking			0.841	0.378***
Study did not require a minimum of 1 year of cessation before included in study				2.802***
Q-statistics	52.62	52.41	51.96	42.77
Wald test of joint significance (Prob > Chi2)	0.08*	0.19	0.15	0.00**

Number of studies: 5; Number of observations: 20; * = significant 90% confidence level, ** 95% confidence level, *** 99% confidence level; a = models differ based on included explanatory variables.

**Figure 2:**
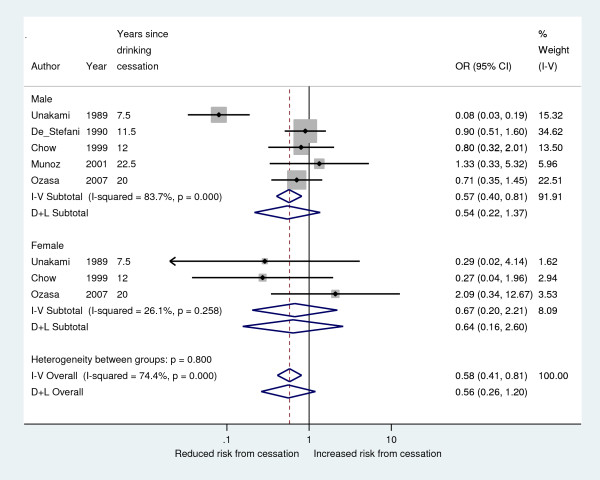
**Risk reduction after alcohol cessation (high vs. current).**

Due to the inconclusive results from the trend meta-analysis estimations above, a so-called “high vs. low” estimation was performed in order to investigate if there was any association between drinking cessation and reduced risk of stomach cancer. Here, the longest drinking cessation group in each study was compared to current drinkers. The results are presented in the forest plot below (Figure 2) and questions if there is a protective effect from drinking cessation, mirroring the meta-analysis trend estimation results. The fixed effect estimate did indicate a protective effect although with a large between-study heterogeneity (about 74% of the variation is attributable to heterogeneity according to the I^2^-statistic). The random effect estimate showed about the same effect size as the fixed estimate, although a much larger confidence interval caused the estimate to be insignificant.

## Discussion and conclusions

The literature review reported several studies that estimated the risk of stomach cancer among former drinkers. All were observational studies with varying results, although more studies indicated an increase in risk for former drinkers compared to current drinkers than showed a reduction in risk. Individual studies also examined the independent effect of alcohol type on risk of stomach cancer [26] and the varying quantities of alcohol historically consumed [26,30] and found no significant effect on stomach cancer controlling for these factors. Only five studies quantified the impact of drinking cessation by duration and presented enough information to allow the dose-risk relationship between time since cessation and risk of disease to be estimated. The meta-analysis, like the systematic review, failed to find a significant effect of alcohol cessation on stomach cancer. This should be contrasted with similar studies of other cancer types where the alcohol-related elevated risk was found to disappear after 16 (oesophageal) and 23 (liver) years following drinking cessation [31,32]. The knowledge base regarding risk decline is thus very low in terms of existing studies. It is not surprising that the current meta-analysis fails to find any significant effects, based on the low number of included studies (and their results) as this affects the power of the test.

Smoking has traditionally been considered a risk factor for stomach cancer [20]. Connected to this is the potential interaction effect between alcohol and smoking. That is, an individual that both smokes and drinks alcohol has a higher risk of disease than purely the sum of the individual risks [20]. This would imply that the beneficial effect of drinking cessation is higher among smokers than non-smokers. The other side of this issue is that drinking cessation has a tendency to lead to smoking cessation [33]. As the current dose-risk estimation was based on summarised data, it was not possible to account for either of these issues in the current study.

There is a concern that individuals who stop drinking do so for health-related reasons (or even suspicion of stomach cancer) [19]. This might bias estimations of the effect of drinking cessation unless it is accounted for by defining a required time period since drinking cessation in order for the individual to be defined as a former drinker. Only Ozasa [25] did not have a required time period. The control for this issue in model 4 of Table 2 shows a markedly higher risk of disease at the time of cessation if a required time period since drinking cessation is not defined, which most likely is an effect of including individuals who stop drinking due to stomach problems. The Chow et al. study [26], on the other hand, showed a large risk increase from drinking cessation in the short term (three years), despite defining a former drinker as someone who had abstained for more than 2 years.

Prior studies have found “former drinker” categories often to be over-represented by former heavy drinkers and alcoholics [34]. If this is the case, the risk of stomach cancer for the cessation group *before* cessation is expected to be higher than for current drinkers. The increase in risk after cessation may therefore be an artefact of poor matching of study groups. There is doubts about how well (some of) the included studies have matched their control and cases groups, although not enough information is available in order to test if any systematic differences exist that may explain why an increase in risk was observed for just quitters compared to current drinkers. In light of the findings in a recent meta-analysis that only heavy drinking is associated with stomach cancer [12], the concern that “former drinkers” are over-represented by former heavy drinkers further complicates the issue. This would mean that the results of the current meta-analysis argue against the reversibility of alcohol-related stomach cancer risk. It is therefore an absolute necessity that future epidemiological studies include and control for the individuals’ level of consumption, not only at drinking cessation but also prior consumption.

Understanding the effect of drinking cessation on the risk of disease is important for many reasons. Several recent studies have estimated the societal cost of alcohol consumption [13,35,36]. Such studies generally give a moment-in-time assessment of the burden of alcohol on society. However, these studies provide little information about what part of the cost could be avoided given effective interventions, a question that requires information on the effect of drinking reduction on alcohol-related disease risk [37]. This information is mostly lacking today, not only for stomach cancer but for all alcohol-related diseases. This research gap also hinders other empirical applications, such as economic evaluations, from being conducted appropriately.

This discussion and the results of the literature review and meta-analysis should be interpreted in relation to the discussion as to whether alcohol increases the risk of stomach cancer in the first place. As mentioned in the introduction, there is no consensus on the potential effect of alcohol, and many studies show different results although the latest meta-analysis finds an effect for heavy but not for moderate drinkers [12]. The rationale for carrying out this study was that a meta-analysis came to the conclusion that alcohol consumption increases the risk of stomach cancer for both men and women, up to a relative risk of 1.32 for the heaviest consumers compared to abstainers [9]. This meta-study in turn was used in the Swedish cost-of-alcohol study [13], and any future estimation of the avoidable cost in Sweden will have to be conducted in relation to this fact, either by excluding stomach cancer or accounting for risk reduction (using these or new relative risk estimates). The evidence summarised in this paper on the effect of drinking cessation on the alcohol-related elevated risk of disease adds further uncertainty. It is our view that the association between level of alcohol consumption and stomach cancer needs to be more robustly shown before further research into the effect of drinking cessation on risk is conducted. However, the general question of the effect of drinking cessation is important, and more information is needed for stomach cancer as well as all other alcohol-related diseases. Future studies should then preferably be longitudinal natural experiments that allows for the causal effect of drinking cessation to estimated.

## Competing interests

The authors declare that they have no competing interests.

## Authors’ contributions

JJ and U-GG designed the study. The literature review and data extraction were initially performed by JB and independently checked and updated by JJ. GH and JJ conducted the meta-analysis. All authors participated in interpretation of the results and the write-up and have approved the final version.

## Pre-publication history

The pre-publication history for this paper can be accessed here:

http://www.biomedcentral.com/1471-2458/13/600/prepub

## Supplementary Material

Additional file 1 **Literature review.**[38-47]. Click here for file
