# Supplementary material for: Time characteristics of the effect of alcohol cessation on the risk of stomach cancer – a meta-analysis
Source: BMC Public Health. 2013 Jun 20;13:600. doi: 10.1186/1471-2458-13-600 (PMC3695890; doi:10.1186/1471-2458-13-600)
Supplement: Additional file 1 — Literature review.[38-47]. [file 1471-2458-13-600-S1.pdf]

Additional file

The literature review identified 19 studies (see **Table A.1**); eight prospective cohort studies and eleven case-control studies. Six studies [32, 38-42] drew their participants from the same cohort, although a different sample size or follow-up period was used. One case-control study [28] recruited participants from the same site as two of the above cohort studies [39-40].

The majority of the studies were conducted in Asia, with Japan the location of ten of these studies, two in South Korea, and one in Taiwan, India, and China respectively. The remaining four studies were conducted in the United States, Venezuela, Uruguay, and Poland. The quality aspects of the identified studies were difficult to assess due to limited information provided in the published articles, beyond what is discussed below.

With three early exceptions, in which the studies were initiated in 1965 [41-42] and 1977 [33], the studies were conducted during the 1980s and 1990s. All of the case-control studies and four of the cohort studies investigated the risk of morbidity, while the remaining four cohort studies investigated mortality risk. Four of the studies analysed cancer risk for stomach sub-sites [2, 10, 28, 33]. Gender-specific results were calculated in 12 of the studies, and five of these studies presented risks for males only. Female-specific calculations were not presented due to uncommon alcohol use among women in three studies [10, 28, 31]. Irregular features of two of the samples should be highlighted: participants in the cohort studies conducted by Inoue *et al* [39] and Kato *et al* [40] were patients who had undergone gastroscopic examination, and the cohort investigated by Sauvaget *et al* [43] consisted of atomic-bomb survivors in Hiroshima and Nagasaki.

In the cohort studies, the sample size ranged from 3,914-44,930 participants. In the case-control studies, the sample size of cases ranged between 152-1,347 participants, and the sample size of controls ranged between 221-1,451 participants. Hospital or clinic controls

were used in five of the studies, and general population or neighbourhood controls were used in five of the studies. One case-control study [44] used both hospital and population controls. Based on the scarce information available, the data appear to have been collected in a similar fashion for cases and controls in most studies, although, in two instances, next of kin interviews were used for some cases due to death or illness [2, 29] and, in some instances, cases were interviewed in the hospital, while controls were interviewed in their homes. In the study that utilised both population and hospital controls [44], cases and hospital controls were interviewed prior to diagnostic procedures and were instructed to provide information regarding their lifestyle during the period before the onset of symptoms; population controls were instructed to provide “usual” diet information. Furthermore, Unakami *et al* [33] reported that clinical information on alcohol consumption was collected from cases, while control patients completed a questionnaire on drinking habits.

Matching according to sex and age among cases and controls was performed in nine of the case-control studies. One study selected controls by random sampling to have a similar age distribution to cases [33]. In addition to sex and age, some studies matched cases and controls according to race, administrative division, time of hospitalization, religion, and mother tongue (**Table A.1**). In the analyses, the following potential confounders were also controlled for in the different studies: smoking status, chewing (areca nut, quid with or without tobacco, or tobacco alone) habit, intake of salty and spicy food, vegetable intake, education, income/socioeconomic status, birthplace, living on a farm, area of residence, family history of cancer, radiation dose, calendar period, and Helicobacter pylori infection (**Table A.2**).

Response rates, which were presented for 14 of the studies, were mixed, with the lowest rate of 27.5% among population controls reported by Hoshiyama & Sasaba [44]. This study, however, also used hospital controls.

The length of time a participant must have abstained from alcohol consumption to be classified as a “former” or “ex” drinker is important when considering the validity of the study results, as discussed in the data extraction section. Eight of the studies provided a clear definition of a “former” or “ex” drinker, although time frames ranged from zero years (stopped at the time of the interview) to five years. For seven of the studies, the definition was not explicitly stated but could in some cases be inferred from background information provided (see **Table A.2**). The OR for stomach cancer among ex-drinkers compared to non-drinkers was calculated in nine of the studies. Six of the studies calculated the relative risk (RR), and one study each calculated the incident rate ratio (IRR) and the death rate. One study only estimated the OR of ex-drinkers compared to current drinkers, while one study did not present an OR/RR for the analysis of alcohol consumption (see **Table A.2**).

Twelve of the studies found no significant increased risk for former drinkers compared to never drinkers (see **Table A.2**). Chow *et al* [29] reported an increased risk only among former drinkers who had stopped drinking within the past five years. The two Korean studies [34, 45] found an increased risk only for women, while three studies [10, 28, 31] found an increased risk for men. Song *et al* [34] and Ji *et al* [10] did not provide a clear definition of a former drinker, although, based on the available information, it may be inferred that Song *et al* [34] classified those who had stopped drinking when baseline data were collected as ex-drinkers, and Ji *et al* [10] classified those who had abstained from drinking for at least one year before the research interview as ex-drinkers. In the case of Shin *et al* [45], nothing can be inferred regarding the definition of a former drinker. Munoz *et al* [31] required an abstinence period of at least five years prior to the interview. Inoue *et al* [28] classified former drinkers as having stopped drinking at the time of data collection, and when ex-drinkers were divided into those who had quit less than one year prior to the interview and those who had quit one or more years before the interview, a significant increased risk was

only observed among the more recent abstainers. Inoue *et al* [28] analysed the risk for cancer of each stomach sub-site, and, among all ex-drinkers, found the highest risk for cardia cancer, followed by middle cancer. However, again, when ex-drinkers where divided based on duration of abstinence, the increased risk remained significant only among the more recent quitters. Ji *et al* [10] also examined risk by stomach sub-site and found that when stomach sub-site was specified, the increased risk remained only for distal cancer but not for cardia cancer. Of the seven studies that demonstrated some increased risk among ex-drinkers, one was a prospective study and six were case-control studies. Only one study [44] showed a significantly reduced risk for ex-drinkers, but only when compared to hospital controls.

Two of the studies provided specific estimates for different levels of consumption. Song *et al* [34] presented separate RRs for former drinkers who had consumed between 12 and 100 g/week or over 110 g/week compared to former drinkers who had consumed less than 12 g/week; the investigators did not find an increased risk for increased weekly level of alcohol intake. Chow *et al* [29] presented separate ORs for former drinkers who had consumed less than 1 drink, 1 to 3 drinks, 3 to 7 drinks or more than 7 drinks weekly and found no increased risk at any level compared to non-drinkers.

Chow *et al* [29] is the only study to have calculated the OR based on beverage type (i.e., beer, wine, and liquor), and the results indicated no increased risk for different types of alcohol. The investigators also presented ORs for different levels of drink-years (number of drinks per week times number of drinking years) and for different ages of initiating alcohol consumption (less than 20, 20-24, greater than 25), with no support for a dose-response trend for either factor.

A related point is whether the studies provided information on the drinking habits of former drinkers, as any observed differences may be due to the fact that the former drinkers may

have consumed higher amounts of alcohol during their drinking years, may have consumed alcohol with greater frequency, or may have started drinking at an earlier age. This information was presented in one of the studies [34]. When the alcohol consumption histories of former drinkers were compared to current drinkers, Song *et al* [34] found that female ex-drinkers who started drinking at a younger age (38.1 vs. 40.7 years of age), consumed greater amounts of alcohol per week (36.5 vs. 16.4g/week), and consumed alcohol for longer periods (24.5 vs. 18.46 years). Male ex-drinkers consumed greater amounts of alcohol per week compared to male current drinkers (99.5 vs. 60.3 g/week) and drank for a longer period (40.6 vs. 33.2 years).

Table A.1 – Key characteristics of studies that have assessed the impact of drinking cessation on stomach cancer

**Study**	**Country and study year(s)**	**Type of study**	**Mortality/ morbidity**	**Age**	**Gender cases (% male)**	**Source of controls**	**Sample size (case/control)**	**Response rate case/control (%)**	**Matched case-control study (matching factors)**
Chen et al. 2000 [46]	Taiwan 1992-1996	Case-Control	Morbidity	29-88	66.4% of cases	Hospital-based controls	152 cases, 497 controls	95.6% cases, 98% controls	Age (±3 years), sex, and time of hospitalization (±2 weeks of the case interview)
Chow et al. 1999 [29]	Poland 1994-	Case-Control	Morbidity	21-79	65.1% of cases	Population Controls	464 cases, 480 controls	90% cases, 87% controls	Age, sex
De Stefani et al 1990 [30]	Uruguay 1985-1988	Case-Control	Morbidity	N/A	100% (for alcohol estimations)	Hospital-based controls	210 cases, 630 controls (smaller sample for alcohol estimations)	100%	Sex and age (±5 years)
Fujino et al. 2002 [38]	Japan 1988-1997	Prospective Cohort	Mortality	N/A	41.7% of participants in analysis, 68.9% of stomach cancer deaths	N/A	Data analyzed for 44,930 participants; 328,030 person-years of follow-up	N/A	N/A
Gajalaksmi & Shanta 1996 [47]	India 1988-1990	Case-Control	Morbidity	N/A	74.0% of cases	Hospital-based controls	388 cases, 388 controls	N/A	Age (±5 years), sex, religion, and mother tongue
Hoshiyama & Sasaba 1992 [44]	Japan 1984-1990	Case-Control	Morbidity	N/A	70.1% of cases	Population and Hospital controls	294(cases)/294 (population controls)/202 (hospital controls)	Of population controls, 1,113/4,052 (27.5%) agreed to participate, additional 150 were excluded, 294 were matched to cases	Population controls matched to cases on sex, age (in 2-year units), administrative division
Inoue et al. 1994 [28]	Japan 1988-1991	Case-Control	Morbidity	N/A	62.9% of cases	Hospital-based controls	668 cases, 668 controls	26,982/28,317 (95.3%) of first-visit outpatients completed questionnaire; 26,426/26,982 (97.9%) provided an adequate response; 668 cancer patients and 668 controls participated	Sex, Age ±2 years, Time of hospital visit ±2 months
Inoue et al. 1996 [39]	Japan 1985-1995	Prospective Cohort	Morbidity	N/A	47.5% of total subjects 73.9% of cases	N/A	5,373 in analysis of cohort, 69 cancer cases	6,226/7,019 (88.7%) of eligible patients provided adequate response to questionnaire, 5,384 (76.7%) remained as original cohort; 5,373 (76.5%) used in analysis	N/A
Ji et al. 1996 [10]	China 1988-1989	Case-Control	Morbidity	20-69	68.5% of cases	Population controls	1,124 cases, 1,451 controls	65.5% cases, 85.8% controls	Age (5 year category), sex
Kato et al. 1992 [40]	Japan 1985-	Prospective Cohort	Morbidity	N/A	47.3% of subjects, 77.8% of cancer cases	N/A	3,914 in analysis; 45 cases of stomach cancer	88.7% of questionnaires collected (6,226/7,019); 5,395 (76.9%) comprised original cohort; 3,914 (55.8%) included in analysis	N/A

Study	Country and study year(s)	Type of study	Mortality/ morbidity	Age	Gender cases (% male)	Source of controls	Sample size (case/control)	Response rate case/control (%)	Matched case-control study (matching factors)
Kono et al. 1985 [41]	Japan 1965-1977	Cohort	Mortality	N/A	100% (only males in study)	N/A	5,139 analysed; 221 cancer deaths; 75 stomach cancer deaths	11,339 responders (49% response rate); 5,477 were followed up; 5,139 were analysed	N/A
Kono et al. 1986 [42]	Japan 1965-1983	Cohort	Mortality	N/A	100% (only males in study)	N/A	5,477 in study population; 5,135 included in analysis; 116 stomach cancer deaths	51% response rate	N/A
Munoz et al. 2001 [31]	Venezuela 1991-1997	Case-Control	Morbidity	Cases> 35	N/A	Neighbourhood controls	292 cases, 485 controls	Cases: 409 identified; 52 did not meet entry criteria and 39 refused= 302/409=73.8%; 10 later excluded Controls: N/A	Age within 5 years and sex
Ozasa 2007 [32]	Japan	Prospective Cohort	Mortality	N/A	66.8% of stomach cancer deaths	N/A	Person years among non-drinkers: 645,420 Person years among drinkers: 604,875 Person years among ex-drinkers: 41,066	N/A	N/A
Sauvaget at al. 2005 [43]	Japan 1980-1999	Prospective Cohort	Morbidity	34-98	38.6% of participants 56.2% of cases	N/A	38,576 total participants 485,575 person-years of follow-up 1,270 cases of gastric cancer	40,349/55,650 answered questionnaire (72.5% response rate) 38,576 included in study	N/A
Shin et al. 2011 [45]	South Korea 2003-2008	Case-Control	Morbidity	>49	69& of cases, 51% of controls	Hospital-based (screening participants)	445/370 cases/controls	N/A	N/A
Song et al. 2008 [34]	Korea 1993-	Prospective Cohort	Morbidity	>64	53.0% of cases; 31.8% of participants	N/A	13,396 in study 151 cancer cases	First collection: 9,026/16,523 (54.6%); Second collection: 6,024/13,926 (43.3%); 13,396 included in study	N/A
Unakami et al. 1989 [33]	Japan 1977-1988	Case-Control	Morbidity	N/A	68.6% of cases	Clinic controls	1347 cases, 221 controls	N/A	N/A
Wu et al. 2001 [2]	U.S.A. 1992-	Case-Control	Morbidity	30-74	Gastric cardia 83.4% Gastric distal 58.9%	Neighbourhood controls	277 gastric cardia, 443 gastric distal, 1,356 control	55% of cases identified, 77% of cases approached (74% gastric cardia, 78% distal gastric)	Gender, race, date of birth ± 5 years

Note: N/A = not available

Table A.2 – Key statistical information from the studies that have assessed the impact of drinking cessation on stomach cancer

**Study**	**Controlling for potential confounders**	**Statistical analysis**	**Time since drinking for being classified as a former drinker**	**OR for former drinkers, compared to NON-DRINKER**	**OR never drinkers**
Chen et al. 2000 [46]	Age, sex, socioeconomic status, and years of schooling	Conditional logistic regression	≥ 6 months before diagnosis	1.4 [0.7-3.1]	N/A
Chow et al. 1999 [29]	Age, education, years lived on a farm, pack-years of cigarette smoking, family history of cancer	Multiple logistic regression	> 2 years	Any alcohol (cmp to abstainers)<br>Men:<br>0.8 [0.4-1.4]<br>2-4 yr 2.2 [1.0-5.1]<br>5-9 yr 0.4 [0.2-1.1]<br>≥10 yr 0.4 [0.2-0.9]<br><br>Women:<br>0.8 [0.4-1.8]<br>2-4 yr 8.0 [1.6-41.4]<br>5-9 yr 0.2 [0.0-1.4]<br>≥10 yr 0.3 [0.1-1.1]	N/A
De Stefani et al. 1990 [30]	Age, sex, residence, smoking duration, wine consumption and vegetable intake	Unconditional logistic regression	Unclear, likely 1 year before interview	Only compared to current drinkers, see below.	N/A
Fujino et al. 2002 [38]	Age	Cox proportional hazards regression analysis	0 year	Men RR 1.16 [0.71-1.87]<br><br>Women RR 0.99 [0.24-4.01]	N/A
Gajalaksmi & Shanta 1996 [47]	Chewing habit, income group, educational level, area of residence	Conditional logistic regression for matched analysis	≥ 6 months before diagnosis	Univariate: 1.9 [1.09-3.39]<br>Multivariate: 1.4 [0.54-3.40]	N/A
Hoshiyama & Sasaba 1992 [44]	Sex, age, administrative division, smoking status, area	Conditional and unconditional logistic regression analyses	Unclear, asked respondents to give lifestyle information from before onset of symptoms	Comparison with population controls RR 0.7 [0.3-1.8]<br><br>Comparison with hospital controls RR 0.3 [0.1-0.8]	N/A

**Study**	**Controlling for potential confounders**	**Statistical analysis**	**Time since drinking for being classified as a former drinker**	**OR for former drinkers, compared to NON-DRINKER**	**OR never drinkers**
Inoue et al. 1994 [28]	Sex	Mantel-Haenszel Method	0 year	Total: 1.87 [1.11-3.15]<br>Cardia: 2.81 [1.21-6.54]<br>Middle: 2.29 [1.12-4.68]<br>Antrum: 1.36 [0.69-2.70]<br><br><1 year after quitting<br>Total: 2.60 [1.09-6.19]<br>Cardia: 3.71 [1.02-13.5]<br>Middle: 3.63 [1.23-10.7]<br>Antrum: 2.16 [0.75-6.25]<br><br>≥1 year after quitting<br>Total: 1.60 [0.87-2.94]<br>Cardia: 2.47 [0.93-6.59]<br>Middle: 1.78 [0.75-4.23]<br>Antrum: 1.06 [0.46-2.45]	N/A
Inoue et al. 1996 [39]	Sex, age, residence	Cox proportional hazards regression model	N/A	RR for gastric cancer compared with subjects without atrophic gastritis 1.87 [0.67-5.22]<br><br>RR for gastric cancer among subjects with atrophic gastritis 1.88 [0.78-4.50	N/A
Ji et al. 1996 [10]	Age, income, education and smoking [cigarettes/day]	Logistic Regression	Unclear, likely 1 year before interview	Males:<br>All cases: 1.91 [1.16-3.15]<br>Cardia: 1.03 [0.40-2.67]<br>Distal: 2.16 [1.27-3.69]	N/A
Kato et al. 1992 [40]	Sex, age, residence	Cox's proportional hazards regression model	N/A	RR for stomach cancer by baseline habit (past alcohol intake) 2.19 [0.78-6.19]	N/A
Kono et al. 1985 [41]	Age and smoking	Mantel-Haenszel and Logistic regression analysis	Likely 0 years (same cohort as used in Kono et al 1986 -- "Men who had drunk in the past and did not currently drink were regarded as ex-drinkers")	Stomach Cancer Death Rates for Ex-drinkers: 170.8 (per 100,000)	Stomach Cancer Death Rates for Non-drinker: 90.9 (per 100,000)
Kono et al. 1986 [42]	Age and smoking	Mantel-Haenszel and Logistic regression analysis based on the Cox proportional hazard model	Likely 0 years ("Men who had drunk in the past and did not currently drink were regarded as ex-drinkers")	RR 1.0 [0.5-2.1]	N/A
Munoz et al. 2001 [31]	Age and SES	Unconditional logistic regression	At least 5 years prior to interview	Compared to non-drinker<br>3.5 [2.0-6.0]<br><br>Compared to 6-10 years since quitting<br>11-15 yr 0.8 [0.2-2.8]<br>16-20 yr 1.3 [0.3-5.3]<br>>20 yr 1.1 [0.3-4.8]	N/A

Study	Controlling for potential confounders	Statistical analysis	Time since drinking for being classified as a former drinker	OR for former drinkers, compared to NON-DRINKER	OR never drinkers
Ozasa 2007 [32]	Age	Hazard ratio	0 year	Men:<br>1.16 [0.86-1.57]<br>Cessation since:<br><5 yr 1.31 [0.76-2.25]<br>5-15 yr 0.77 [0.41-1.44]<br>15+ yr 0.73 [0.35-1.5]<br><br>Women:<br>1.44 [0.71-2.93]<br>Cessation since:<br><5 yr N/A<br>5-15 yr 2.45 [0.78-7.68]<br>15+ yr 2.09 [0.51-8.45]	N/A
Sauvaget at al. 2005 [43]	City, sex, sex-specific age, calendar period, education, radiation dose	Poisson regression	N/A	Incident Rate Ratio (IRR) 1.09 [0.78-1.51]	N/A
Shin et al. 2011 [45]	Sex, age, Helicobacter pylori infection, smoking, education	Logistic regression	N/A	All<br>1.68 [1.07-2.64]<br><br>Men<br>1.29 [0.77-2.17]<br><br>Women<br>3.07 [1.14-8.28]	N/A
Song et al. 2008 [34]	Smoking and intake of salty and spicy food	Cox's proportional hazard model	Unclear, likely 0 year (states participants classified into never, former, and current *smoker* according to status of the baseline)	Men RR 1.00 [0.50-1.98]<br><br>Women RR 2.85 [1.11-7.32]	N/A
Unakami et al. 1989 [33]	Gender and Age	Cornfield and Haenszel method; Mantel and Haenszel test for significance	At least 5 years prior to surgery		N/A
Wu et al. 2001 [2]	Smoking status, age, sex, race, birthplace, and education	Unconditional Logistic Regression	Unclear, likely status 1 year before date of diagnosis	Gastric cardia: 0.91 [0.6-1.4]<br>Distal gastric: 0.85 [0.6-1.2]	N/A

Note: N/A = not available
